# The New York Institute of Stomatology

**Published:** 1902-06

**Authors:** 

**Affiliations:** The New York Institute of Stomatology


### Reports of Society Meetings.

### THE NEW YORK INSTITUTE OF STOMATOLOGY.

A MEETING of the Institute was held at the office of Dr. E. A. Bogue, No. 63 West Forty-eighth Street, New York, on Wednesday evening, February 12, 1902, the President, Dr. J. Morgan Howe, in the chair.

The minutes of the last meeting were read and approved.

The regular order of business was reversed and Dr. Park Lewis's paper, the formal paper of the evening, was presented before the communications on theory and practice.

(For Dr. Lewis's paper, see page 393.)

### DISCUSSION.

*Dr. Wendell C. Phillips.*—I am sure it has been a great pleasure, first, to be a guest of this society, and secondly, to listen to Dr. Lewis's paper. It will be impossible to discuss all the points that have been touched upon, and it has seemed to me that I could spend the time better in discussing those points which especially interest your profession. I would like to preface my remarks by stating something that perhaps many of you would hardly believe, that, on an average, one child in every eight or ten has adenoid vegetations in the vault of the pharynx or hypertrophied tonsils. The proportion is a very large one, and when we come to consider this proportion with the very few who present dental deformities, it seems to me that it rather opposes the theory outlined by Dr. Lewis. About two years ago I read a paper before the Odontological Society, the subject of which was a discussion of "The Sequelæ of Adenoids in the Vault of the Pharynx." At that time I made a considerable study of deformities. A dentist came to my clinic and made casts of the cases as they came in. Out of about twelve cases there was only one that presented a dental deformity. Deflection of the septum naturally accompanies a high arched vault, but I think it is more frequently the result of external violence. The narrow jaw, high arch, narrow face, and the peculiar facial expression are only seen when these cases are very marked and extreme in character and when the adenoids appear very early in life. I do not think you will have very much deformity when the adenoids begin to develop at seven or nine years of age, as is often the case. That children are born with adenoids I believe to be of very rare occurrence. I believe that heredity enters into the case more than the congenital condition. I have seen the same general characteristics of hypertrophy in mother and child, but I do not think it necessarily follows that the child was born with the enlarged tonsils.

In regard to the general effect upon the child, on account of difficult breathing and lack of oxygenation of the blood we find the pigeon chest, and we may get rhachitis and lack of development along various lines. These results are rather more those due to improper oxygenation than anything else. I do not mean to be understood as saying that I do not believe adenoids are the cause of high arched palates, but I believe it to be the exception rather than the rule. I think Dr. Lewis's statement in regard to the ear is absolutely correct. Almost without exception, where we find acute suppuration of the middle ear in a child, that child has adenoids. I have rarely known it to fail. For this reason, if for no other, operation should be performed. The reason for this persistent ear complication is easily explainable. The adenoids fill up the space between the openings of the Eustachian tubes. Then when there is a cold the infection by various means gets into the Eustachian tube and along that into the middle ear. There is a common expression among medical men that running ears in children are due to dentition. I think it is pretty well understood now that dentition has no more to do with running ears than stone in the bladder, and that you never have discharging ears in children unless you introduce infection by way of the Eustachian tube.

Regarding the disappearance of this lymphoid tissue at puberty, no doubt it does disappear to a certain extent at that time, but that is no argument against the importance of their removal because before that time the damage may have been done. If you retard the physical development of a child you will retard his mental development, and this condition certainly does very much retard the physical development.

As to the eye, it might seem when you first consider it that it is a rather far cry. However, I am not going to discuss that, as Dr. Davis is much better able to do so than I. The only way that it would seem to me reasonable that the eye could be affected would be by the effect on the general nutrition.

In order that I may impress upon you the degree this condition may reach in retarding the development of a child, I have in mind a patient who suffered with adenoids in a severe form. He was never able to breathe through the nose. At the age of sixteen years he was a little "runt," and had never seen a healthy day in his life. A thorough removal of the growths resulted in almost a miracle. In one year he gained fifty pounds in weight. From a boy he shot into a man with great rapidity. In every instance increases in weight have followed this operation.

*Dr. A. E. Davis.*—Dr. Lewis has said that the adenoids in the throat affect the eye. I agree with him to the extent that they do so in a general way, but not directly as they do in the ear. As you know, in the eye itself we find no lymphatics. We do, however, find lymphatic spaces. These lymph spaces inside of the eye connect through the sheath of the optic nerve with the lymph spaces in the brain. Nevertheless, these adenoid growths do to a certain extent affect the eye. Regarding the case Dr. Lewis mentioned of a conjunctivitis not being relieved until the adenoids were removed from the pharynx, I have seen such cases myself. I have seen cases where a conjunctival inflammation resisted all treatment until adenoids in the pharynx were removed.

As far as the development of the eye is concerned, and these conditions causing hypermetropia and squint, I think this is rather due to the fact that most of the eyes of children are hypermetropic until they are ten years of age. So the fact that we find hypermetropia in these cases of adenoid growths is no indication that there is any relation between the two. Myopic children are very rare, and for that reason we would observe very few myopic children with adenoid growths. There are cases reported where squint has disappeared after this operation, but no doubt it is due to the increased nutrition. Again we should not be too sure of the direct actual relation between the two, because in a great many cases squinting eyes are outgrown. With proper correction of the refractive error about thirty per cent. of squinting eyes can be cured without operation. I wish every oculist could have a reprint of this paper, especially those who are so prone to cut muscles. It is my opinion, and in this I agree most thoroughly with Dr. Lewis and the rest of you, that in cases of muscle insufficiency nine-tenths of the children have adenoid growths which tend to reduce nutrition. We have oculists, I am sorry to say, who try to balance every muscle of the eye, giving no thought to the general nutrition. I think ninety-nine per cent. of these cases can be cured with proper attention to the general nutrition. In regard to the experiment of sewing up the rabbit's nose and causing hypermetropia, it is a well-known fact that animals, without exception, are born hypermetropic and remain so. Even in mankind we do not become myopic until we are civilized. All uncivilized races are hypermetropic. It is the rarest thing in the world to find the ideal, the emmetropic eye. I believe then that the direct effect on the eye of these lymphatic enlargements in the pharynx is not marked, but indirectly by affecting the general health and nutrition these growths may and often do have a bad effect on the eyes.

*Dr. Haskin.*—I was very much interested in what Dr. Phillips said regarding adenoids in very young children. I have examined several hundred cases in hospitals, and see these growths constantly in children from one to two years of age, and where operation is attended with great relief. Dr. Bogue asked me to speak more especially with reference to adenoids in connection with cleft palate. There are rarely ever cases of cleft palate without adenoids, but as cleft palate is a failure of union in utero this condition can hardly be ascribed to the adenoids. I think in many children where adenoids occur, they are often a symptom. A great many times it is due to improper feeding of the child by the parents. When the child is small, as soon as it begins crying they give it the bottle, and by the time it is eight or ten months of age they give it everything under the sun. That undoubtedly will produce some disturbances in the digestive apparatus which will naturally be followed by a congestion of the lymphatics of the mucous membrane.

Dentition I do not believe has a great deal to do with the production of the middle ear diseases, but at this time, especially in the children of the poor, the mouth is in a very much more inflamed condition and attacks of stomatitis are more frequent, and any inflammation of the mucous membrane of the mouth may extend to the Eustachian tube. I thank Dr. Lewis for his most interesting paper, and you, gentlemen, for having had this opportunity of listening to it.

*Dr. Stuart Close.*—I would like to ask whether, in the experience of the lecturer, there is not a recurrence of the adenoids after operation, and in about what percentage of cases they recur?

*Dr. C. A. Brackett.*—Just a word to express my appreciation for what I have gained this evening. I have known enough of this subject for some years to be greatly interested in it. I made the journey from Newport solely to be present here, and I feel fully repaid for the effort.

*Dr. Davenport.*—While it is no doubt true, as Dr. Phillips has said, that the disturbance of the bony formation and the peculiarity of mouth-breathing are present only in a small proportion of cases where adenoids are to be found, I think the proportion is large enough for dentists to take upon themselves the responsibility, which I consider great, of always being on the lookout for this condition. It has come to my attention that the dentist is often the first to recognize the presence of adenoids in sufficient amount to interfere with the nutrition of the child. It seems to me that this matter is well brought up at this time, to emphasize our duty to our patients, to increase our watchfulness in diagnosing these cases. While it may not be possible for us at a glance to say "this child has adenoids," the presence of the rat-like face, the peculiar expression accompanying mouth-breathing or the pinched and strained appearance of the face usually present,—one or more of these symptoms being recognized should cause us to advise the taking of the child to a specialist for examination.

*Dr. Lewis.*—I will summarize very briefly what I have to say. If you will recall my paper I think you will remember I distinctly stated that the crowded teeth and high palate were by no means always present. You will find very many cases in which there are perfectly normal jaws.

Of course, if the general nutrition of the body suffered, the eye would suffer with the rest, but in the experiments to which I referred the closing up of one nostril was accompanied by hypermetropia in the eye on that side *only,* and not in both eyes, as would occur from a disturbance in general nutrition. Dr. Davis mentioned the absence of lymphatics in the eye except in the conjunctiva. Follicular conjunctivitis is the only disease of the eyes which I have seen as a result of adenoids. I will say, in answer to Dr. Close's question as to how many cases recur after removal, that they do recur, but not frequently. In a large number of cases an operation ends the matter; in some, as after amputation of the tonsils, the remaining structures again hypertrophy. I am very much obliged, gentlemen, for the courtesy I have received.

*Dr. C. O. Kimball.*—I move you, Mr. President, that the most hearty thanks of the Institute be tendered Dr. Lewis and also Drs. Phillips, Davis, and Haskin for so kindly meeting with us to-night and contributing to our instruction and pleasure.

Motion seconded and unanimously carried.

### COMMUNICATIONS ON THEORY AND PRACTICE.

Dr. Davenport presented a letter from Dr. E. H. Raymond.

*Dr. E. H. Raymond.*—There are two things my brethren may find helpful from which "my people" are deriving benefit. One is a compound of milk of magnesia and precipitated chalk, for erosions, made for me by the Charles H. Phillips Chemical Company, 128 Pearl Street. It is made into a paste, and if applied with the finger around the necks of the teeth before retiring, it will be found to remain longer than any preparation yet tried.

A piece of muslin, or thin cloth cut into narrow strips, on one side of which is spread the compound, if placed under the upper lip, will be found effectual in very "wet mouths." To be applied at night.

The other is for obtunding sensitive dentine by the use of nitrate of silver. Add equal parts of powdered nitrate of silver and eucaine (or cocaine), and make into a paste with carbolic acid. Dry the cavity to be treated, saturate it with carbolic acid, and then place the above compound in it and seal it up with soft gutta-percha. I use an asbestos mat to carry the compound. There will be no pain from the application, as in the case of nitrate of silver alone, and you can excavate and fill without pain at a future sitting.

For supersensitive dentine it is the best thing I have ever used, as when properly manipulated not one particle of pain need be given from beginning to end of an operation.

I even use it in front teeth where cavities are accessible, cutting out the stain. A second application may be necessary in many cases after superficial decay is removed.

You all know the good effect of silver nitrate on dentine.

Dr. Bogue presented a device in the shape of a rubber dam holder, patented by Dr. Knowles, of San Francisco, in order that it should not be seized by any manufacturing concern, but should be at the service of dentists everywhere.

Also an efficient nozzle affixed to the tubes of ethyl chloride, to be used in obtunding sensitive dentine by means of which a series of jets can be thrown into the cavity by pressing the thumb on a lever.

Also a model of a rather peculiar case presenting but three lower incisors, with the remains of temporary teeth separating the bicuspids on the right.

*Dr. E. A. Bogue.*—I would like to know from Dr. Lewis if he considers the condition which we have heard so much about this evening influential in producing the narrowing of the jaws.

*Dr. Lewis.*—It has seemed to me quite possible that it might contribute, but I do not believe by any means that this alone is the reason for the contour of the face.

*Dr. H. W. Gillett.*—Dr. Hamilton, of Boston, in writing me some weeks ago about another matter, mentioned a tooth-brush which he had devised. Dr. Hamilton's idea is that in many cases the gums are injured by brushing, and in order to obviate this he has devised this brush, which consists of alternate rows of bristles and badger hair, the hair being longer than the bristles, which serves to keep the hair from matting down. Dr. Hamilton feels that many cases of recession are due to too vigorous brushing, and he has seen much benefit by prescribing this brush.

*Dr. F. Milton Smith.*—I have been polishing teeth a great deal during the past few months, and while I have not made new teeth to grow where there were no teeth, I am quite sure I have improved the condition of the old ones, and in cases of pyorrhœa this polishing has done wonders in some instances. What I want to call especial attention to is this little simple port-polisher, which is made by drilling a hole in the end of an instrument having a heavy shank. The hole is made sufficiently large so that the small-sized orange-wood sticks furnished by the depots will fit tightly. I have a number of these instruments, each differing from the others in the angle at which the hole is drilled, obviating the necessity of changing the points so frequently as when a single instrument having a changeable angle is used. Having half a dozen of these ready at hand when I desire to polish the teeth I find economizes my time.

*Dr. Gillett.*—The old Cogswell file carriers make very handy polishers.

*Dr. Brackett.*—I can endorse the efficiency of the Cogswell carrier. The stick can be turned at any angle. It is very simple and convenient.

*Dr. Kimball.*—Sticks for these polishers, maple and cedar, can be obtained here in New York from piano-action makers, Wessel, Nickel & Gross, 457 West Forty-fifth Street. They are perfectly finished, $\frac{7}{32}$ of an inch in diameter: the maple sticks can be used for wedging and polishing and the cedar for polishing.

While I am on the floor I want to emphasize the method of extirpating a pulp by direct anæsthesia, either by eucaine or cocaine. The subject has been referred to before, having been advocated by Dr. Briggs, of Boston, and others. I have used it a good many times. I recently had an illustration of its efficacy. For the benefit of those who do not use it at all, I would like to explain its *modus operandi.* In one case, for the devitalization of a superior wisdom-tooth, I applied arsenic, with the result that in about three hours the patient returned, the pain being almost unbearable. The tooth was one that could not readily be reached by a hypodermic syringe, but by closing the cavity with gutta-percha with the exception of a small opening I fitted to this opening a conical plug of soft rubber, through which I passed the needle of the hypodermic. By holding this plug very firmly in place with an instrument like a two-tined fork, I was able to close the opening so as to get a minim or so of a four per cent. solution of eucaine injected under strong pressure. There was immediate cessation of pain, although the tooth had been aching violently.

I was then able to remove the pulp without pain. The same thing occurred this week in the case of an upper first molar. In these cases it is necessary to be pretty thorough in the operation, removing the pulp clear to the apical foramen, else there may be pain afterwards. In these cases I have mentioned relief from pain was immediate and final.

Adjourned.

FRED. L. BOGUE, M.D., D.D.S.,
*Editor The New York Institute of Stomatology.*

